# Association of Social Support During Adolescence With Depression, Anxiety, and Suicidal Ideation in Young Adults

**DOI:** 10.1001/jamanetworkopen.2020.27491

**Published:** 2020-12-04

**Authors:** Sara Scardera, Léa C. Perret, Isabelle Ouellet-Morin, Geneviève Gariépy, Robert-Paul Juster, Michel Boivin, Gustavo Turecki, Richard E. Tremblay, Sylvana Côté, Marie-Claude Geoffroy

**Affiliations:** 1 McGill Group for Suicide Studies, Department of Psychiatry, Douglas Mental Health University Institute, Montreal, Quebec, Canada; 2 Department of Educational and Counselling Psychology, McGill University, Montreal, Quebec, Canada; 3 School of Criminology, University of Montreal, Montreal, Quebec, Canada; 4 Research Center of the Montreal Mental Health University Institute, Montreal, Quebec, Canada; 5 Department of Social and Preventive Medicine, University of Montreal, Montreal, Quebec, Canada; 6 Department of Psychiatry and Addiction, University of Montreal, Montreal, Quebec, Canada; 7 School of Psychology, Laval University, Quebec City, Quebec, Canada; 8 Department of Psychology, University of Montreal, Montreal, Quebec, Canada; 9 CHU Ste-Justine Research Centre, Montreal, Quebec, Canada; 10 INSERM 1219 Bordeaux Population Health, Bordeaux, France

## Abstract

**Importance:**

Mental health problems are common during the transition from adolescence to young adulthood. Although perceived social support and mental health problems have been shown to be concurrently associated, longitudinal studies are lacking to document the directionality of this association, especially in emerging adulthood (late teens to late 20s).

**Objective:**

To test whether social support in emerging adulthood protects against later depression, anxiety, and suicidal ideation and suicide attempts after adjusting for a range of confounders, including prior mental health problems and family characteristics.

**Design, Setting, and Participants:**

This population-based cohort study included 1174 participants from the Quebec Longitudinal Study of Child Development. Participants underwent yearly or biennial assessment (starting from age 5 months to age 20 years). Data were collected from March 16, 1998, through June 1, 2018.

**Main Outcomes and Measures:**

Self-reported perceived social support was measured at age 19 years using the 10-item Social Provision Scale. Mental health problems, including depressive and anxiety symptoms as well as suicidal ideation and attempts, were measured at age 20 years. Social support and mental health problem raw scores were converted to *z*-scores to ease interpretation. Depressive and anxiety symptoms were categorized using validated cutoffs to determine clinical significance.

**Results:**

The study consisted of 1174 participants (574 female [48.89%] and 600 male [51.11%] individuals). Emerging adults with higher levels of perceived social support at age 19 years reported fewer mental health problems 1 year later, even after adjusting for a range of mental health problems in adolescence at ages 15 and 17 years (eg, depressive and anxiety symptoms and suicidal ideation and attempts) and family characteristics (eg, socioeconomic status and family functioning and structure). Higher perceived social support was associated with fewer symptoms of depression (β = −0.23; 95% CI, −0.26 to −0.18; *P* = <.001 and odds ratio [OR], 0.53; 95% CI, 0.42-0.66 for severe depression) and anxiety (β = −0.10; 95% CI, −0.15 to −0.04; *P* < .001 and OR, 0.78; 95% CI, 0.62-0.98 for severe anxiety). Higher perceived social support was associated with a lower risk for suicide-related outcomes (OR, 0.59 [95% CI, 0.50-0.70] for suicidal ideation and OR, 0.60 [95% CI, 0.46-0.79] for suicide attempts).

**Conclusions and Relevance:**

In this cohort study, emerging adults who perceived higher levels of social support reported experiencing fewer mental health problems 1 year later. These findings suggest that perceived social support may protect against mental health problems during the transition into adulthood, even in those who experience mental health problems in adolescence. Leveraging social support in prevention and treatment options may protect against mental health symptoms during this transition period.

## Key Points

**Question:**

Is perceived social support associated with fewer mental health problems during the transition from adolescence to young adulthood?

**Findings:**

In this cohort study of 1174 adults aged 19 to 20 years, perceived social support was found to be statistically significantly associated with fewer depressive and anxiety symptoms, and suicide-related outcomes at 1-year follow-up even after accounting for key confounders, including prior mental health problems in adolescence.

**Meaning:**

Findings of this study suggest that leveraging social support may play an important role in the prevention and treatment of mental health problems.

## Teaser

This cohort study assesses whether perceived social support during adolescence is associated with less depression, anxiety, and suicidal ideation and fewer suicide attempts in young adults.

## Introduction

Perceived social support refers to the subjective availability of care and assistance received from social relationships and it is characterized by emotional support (eg, expression of empathy), instrumental support (eg, help with household tasks) and informational support (eg, financial advice) that can be provided from various sources, such as friends or family.^1^

Findings of systematic reviews^2,3^ and a meta-analysis^4^ have suggested that individuals who perceive greater social support experience fewer depressive symptoms. However, most of the evidence is based on cross-sectional studies that did not control for prior mental health problems (MHPs), providing no insight into directionality of the association between perceived social support and MHPs. This information gap is problematic because individuals who previously experienced MHPs may be more likely to perceive a lower level of social support and to have smaller social networks of lesser quality, resulting in inflated effect sizes.^5,6^ In addition, many studies use social support measures that lack strong psychometric properties.^2,6^

Social support may be especially important during emerging adulthood (late teens to late 20s), a period simultaneously characterized by major changes in social roles and responsibilities (eg, living independently, transitioning from mandatory education to college or the workforce, establishing romantic relationships^7^) and increased incidence of MHPs.^8,9^ Importantly, MHPs in youth may be associated with lifelong consequences such as lower educational attainment, unemployment, and chronic MHPs.^10,11,12,13^ The evidence regarding associations between perceived social support and MHPs in emerging adulthood remains limited. Few longitudinal studies have examined the role of social support on MHPs in emerging adulthood, while accounting for baseline and/or previous MHPs.^14,15,16^ In 2 longitudinal studies, social support from a non-specified source was found to be protective against depression.^14,15^ However, parental social support was found to be protective against depression only when instilled earlier on in development.^17,18^ Evidence remains scarce for other salient and clinically significant MHPs, including generalized anxiety^17^ and suicidal ideation,^16^ with no studies examining the protective role of social support for suicide attempts during emerging adulthood. Because well-established sex-based differences exist across MHPs, it is imperative to investigate potential sex-based differences in perceptions of social support and the extent to which male and female individuals may benefit differently from social support.

Using a large population-based cohort from the Canadian province of Québec, we aimed to assess whether individuals who perceive higher levels of social support (from a non-specified source) in emerging adulthood have a reduced risk for later MHPs, including depression, anxiety and suicidal ideation and attempts, 1 year later. Because emerging adults may have already exhibited MHPs prior to this transitional life stage, we accounted for a range of MHPs during adolescence and family characteristics. To our knowledge, this study is the first to investigate the association of perceived social support with a range of common MHPs simultaneously within a contemporary population-based cohort of emerging adults (aged 19-20 years) while taking into account previous MHPs and family characteristics to clarify the directionality of these associations.

## Method

### Participants

The Quebec Longitudinal Study of Child Development (QLSCD) is an ongoing population-based cohort that includes 2120 participants born from 1997 through 1998 in Quebec. The QLSCD is conducted by the Institut de la Statistique du Québec.^19^ This cohort study was approved by ethical committees of the Québec Institute of Statistics of the CHU Sainte-Justine Hospital Research Centre and participant written informed consent was obtained. We followed the Strengthening the Reporting of Observational Studies in Epidemiology (STROBE) reporting guideline.^20^

Of the 1680 individuals contacted at the 20-year data collection point, 1235 responded, and 1174 provided information on social support at age 19 years and MHPs at age 20 years (56% of the original 2120 participants). Similar to previous studies that used this cohort, we selected comparison variables that have the potential to identify the most vulnerable participants and, in turn, those most likely to be lost at follow-up.^21,22^ Participants who were underrepresented were more likely to be male, to be of non-Canadian ancestry, to have a younger mother at birth and/or with depressive symptoms, to come from a nonintact family, to have parents with a low socioeconomic status at age 5 months, and to have higher externalizing symptoms at age 29 months (eg, cannot sit still or is agitated) as measured by 10 items from the Behavior Questionnaire,^23^ with scores ranging from 0 to 18 and high scores indicating higher levels of externalizing symptoms (eTable 1 in the Supplement). To minimize bias attributable to such differential attrition, we applied inverse probability weighting based on variables conditional to attrition in all analyses.

### Measures

#### Perceived Social Support at Age 19 Years

Perceived social support was assessed using the 10-item Social Provision Scale (SPS-10^24^), a shortened version of the original 24-item scale,^25^ which measures dimensions of attachment, social integration, reassurance and worth, reliable alliance and guidance based on statements (eg, “there is someone I can talk to about important decisions in my life”) rated on a Likert scale (range, 0 = strongly disagree to 4 = strongly agree). The SPS-10 is a valid and reliable measure of perceived availability of social support (Cronbach α = .89).^24,26^ All SPS-10 scores were converted to *z* scores, with higher scores indicating higher levels social support.

#### Mental Health Problems at Age 20 Years

Depressive symptoms experienced during the past week were assessed using the Centre for Epidemiological Studies Depression scale (CES-D) short-form,^27^ including 12 statements^28,29^ (eg, “I felt depressed”) rated on a Likert scale from 0 = rarely/none of the time to 3 = most/all of the time. Scores ranged from 0 to 36, with higher scores representing increasing symptom severity. The scale has good internal reliability (Cronbach α = .85). Anxiety symptoms experienced during the past 2 weeks were assessed using the Generalized Anxiety Disorder 7-item scale^30^ (GAD-7), with statements (eg, “Feeling nervous, anxious or on edge”) rated from 0 = not at all to 3 = nearly every day. Scores range from 0 to 21, with higher scores representing increasing symptom severity. The GAD-7 is a reliable measure of anxiety symptoms (Cronbach α = .89).

Serious suicidal ideation was assessed with the following question: “In the past 12 months, did you ever seriously think of attempting suicide?” If the answer was affirmative, they were asked how many times they attempted suicide in the last 12 months. Both of these variables were dichotomized as 0 or ≥1.^31^

### Adolescent Confounders

We measured confounders at ages 15 and 17 years and averaged the scores across ages, unless otherwise indicated. Several factors were identified as having associations with both the MHPs of interest (depressive and anxiety symptoms and suicidal ideation and attempts) and perceived social support (eTable 2 in the Supplement). These factors included sex; family socioeconomic status assessed as an aggregate of parental occupation, parental education level, and annual gross income^32^; family functioning (ie, communication, problem resolution, and control of disruptive behavior assessed using the 7-item McMaster Family Assessment Device^33^ [scores range from 0-10, with higher scores indicating more family dysfunction]); and family structure at age 17 years (biological parents, blended family, single parent), as well as MHPs in the last 12 months (measured using the Mental Health and Social Inadaptation Assessment^34^), including for depressive, generalized anxiety, oppositional and/or defiant, inattention or hyperactivity, and conduct disorder symptoms. Subscales for these symptoms are scored as follows: 0 = never true, 1 = sometimes true, 2 = always true. Higher scores represent increasing symptom severity. Suicidal ideation and suicide attempts were measured using the same instruments at age 20 years.

### Statistical Analyses

All statistical analyses were conducted using IBM SPSS, version 26. Prospective associations between perceived social support and MHPs in emerging adulthood were estimated using linear or binary logistic regressions. The first model was adjusted for sex only, whereas the second model was additionally adjusted for adolescent confounders, including MHPs (depressive, anxiety, oppositional/defiant, inattention/hyperactivity and conduct disorder symptoms and suicidal ideation or attempts) and family characteristics (socioeconomic status, family functioning and structure). No sex-based differences were found in social support interactions across prospective associations; thus, we present regression results with sexes combined. Missing data for confounders ranges from 6% (MHPs) to 18.3% (family structure at 17 years). To avoid further loss of data, missing data on confounders were imputed using multiple imputation by chained equations,^35^ and analyses were conducted across 100 imputed data sets (N = 1174). Patterns of results based on multiple imputations were comparable to those based on maximum available cases (eTables 3 and 4 in the Supplement).

Using multinomial logistic regressions, we further estimated the clinical significance of prospective associations between social support and depressive and anxiety symptoms by taking into consideration symptom severity based on validated cutoff values on the CES-D and GAD-7 scales. No or minimal symptoms had scores ranging from 0 to 11 for depression and from 0 to 9 for anxiety on the CES-D and GAD-7 scales, respectively, moderate symptoms had scores ranging from 12 to 20 for depression and 10 to 14 for anxiety, and severe symptoms had scores ranging from 21 to 36 for depression and 15 to 21 for anxiety.^29,30^

## Results

The total sample consisted of 1174 participants, including 574 female (48.89%) and 600 male (51.11%) individuals. Social support was measured at age 19 years and mental health outcomes were measured at age 20 years. The descriptive statistics on key variables are presented in Table 1. Overall, male individuals perceived significantly less social support than female individuals at age 19 years. Mental health problems were assessed at age 20 years, and of the total sample, 289 individuals (24.6%) reported moderate depressive symptoms and 67 (5.7%) reported severe symptoms. One hundred individuals (8.5%) reported moderate anxiety symptoms, and 61 (5.2%) reported severe symptoms. The prevalence rates for suicidal ideation and attempts were 10.3% (121 of 1174 individuals) and 2.5% (30 of 1174 individuals), respectively. Patterns of depressive and anxiety symptoms were generally higher in female than in male individuals. This pattern is present in continuous scores in male and female individuals (mean score, depressive symptoms: 8.14 vs 10.11; anxiety symptoms: 3.52 vs 5.60) as well as in the proportion with moderate (depression: 18.3% [110 of 600] vs 31.2% 179 of 574]; anxiety: 3.8% [23 of 600] vs 13.4% [77 of 574]) and severe symptoms (depression: 4.3% [26 of 600] vs 7.1% [41 of 574]; anxiety: 3.8% [23 of 600] vs 6.6% [38 of 574]) (Table 1). No sex-based differences were observed for suicide-related outcomes. Additionally, correlations between adolescent confounders and the key variables including social support at 19 years and MHPs at age 20 years are described in eTable 2 in the Supplement. Of note, all MHPs at ages 15 and 17 years, except suicide attempts, were significantly correlated with social support at age 19 years (Pearson *r* range, −0.10 to −0.20; *P* < .05) and MHPs at age 20 years (Pearson *r* range, −0.08 to 0.45; *P* < .05).

**Table 1.** Descriptive Statistics for Social Support and Mental Health Problems and Suicide-Related Outcomes at Age 20 Years Based on Weighted Values^a^

	Total (N = 1174)	Males (n = 600)	Females (n = 574)	*P* value for sex differences^b^
Social support, mean (SD)^c^	26.70 (4.05)	26.36 (4.27)	27.06 (3.80)	.003
Depressive symptoms, mean (SD)^c^	9.10 (6.40)	8.14 (6.21)	10.11 (6.44)	<.001
Depression, No. (%)				<.001
Moderate symptoms^d^	289 (24.6)	110 (18.3)	179 (31.2)	NA
Severe symptoms^e^	67 (5.7)	26 (4.3)	41 (7.1)	NA
Anxiety symptoms, mean (SD)^c^	4.53 (4.64)	3.52 (4.27)	5.60 (4.79)	<.001
Anxiety, No. (%)				<.001
Moderate symptoms^d^	100 (8.5)	23 (3.8)	77 (13.4)	NA
Severe symptoms^e^	61 (5.2)	23 (3.8)	38 (6.6)	NA
Suicidal ideation, No. (%)	121 (10.3)	61 (10.2)	60 (10.5)	.87
Suicide attempts, No. (%)	30 (2.5)	15 (2.5)	15 (2.6)	.90

Abbreviaton: NA, not applicable.

^a^ Data were compiled from the final master file of the Québec Longitudinal Study of Child Development (1998–2018), Québec Government, Québec Statistic Institute.

^b^ Based on *t* tests or χ^2^ (none, moderate, and severe).

^c^ Raw scores with a mean (SD) of 0 (1).

^d^ Moderate symptoms were defined by Centre for Epidemiological Studies Depression scale scores of 12 to 20, with a maximum score of 36 and Generalized Anxiety Disorder 7-item scale scores of 10 to 14, with a maximum score of 21.

^e^ Severe symptoms were defined by Centre for Epidemiological Studies-Depression scale scores of 21 or higher and Generalized Anxiety Disorder 7-item scale scores of 15 or higher.

### Associations Between Social Support and MHPs

Prospective associations between perceived social support at age 19 years and MHPs at age 20 years, are shown in Table 2. Emerging adults who perceived a higher level of social support reported fewer depressive and anxiety symptoms 1 year later, after adjustment for sex and all remaining confounders, including previous MHPs. The magnitude of these associations appears stronger for depressive symptoms (β = −0.23 [95% CI, −0.26 to −0.18]; *P* < .001) compared with anxiety symptoms (β = −0.10 [95% CI −0.15 to −0.04]; *P* < .001) in fully adjusted models. Individuals who perceived more social support were also less likely to report suicidal ideation (vs those with no suicidal ideation) and less likely to attempt suicide (vs no attempts), and these associations remained even after adjusting for the potential confounders, including previous MHPs and suicidal ideation and attempts. Every SD increase in social support was associated with a 41% lower odds for suicidal ideation (odds ratio [OR], 0.59; 95% CI, 0.50-0.70) and 40% for suicide attempts (OR, 0.60; 95% CI, 0.46-0.79).

**Table 2.** Associations Between Social Support at Age 19 Years and Mental Health Problems and Suicide-Related Outcomes at 20 Years (N = 1174), Imputed Values^a^

Model	Depressive symptoms		Anxiety symptoms		OR (95% CI)	
	β (95% CI)	*P* value	β (95% CI)	*P* value	Suicidal ideation	Suicide attempts
Social support adjusted for sex	−0.32 (−0.37 to −0.26)	<.001	−0.18 (−0.23 to −0.12)	<.001	0.56 (0.48 to 0.66)	0.56 (0.45 to 0.70)
Social support adjusted for all other confounders^b^	−0.23 (−0.26 to −0.18)	<.001	−0.10 (−0.15 to −0.04)	<.001	0.59 (0.50 to 0.70)	0.60 (0.46 to 0.79)

Abbreviation: OR, odds ratio.

^a^ Data were compiled from the final master file of the Québec Longitudinal Study of Child Development (1998–2018), Québec Government, Québec Statistic Institute.

^b^ The fully adjusted model is additionally adjusted for mental health problems in adolescence (ages 15-17 years), including anxiety, depression, suicidal ideation/attempts, attention deficit/hyperactivity-impulsivity, oppositional defiant and conduct symptoms, and for family characteristics in adolescence, including socioeconomic status, family functioning, and family structure (at age 17 years).

### Associations Between Social Support and Clinical Thresholds of MHPs

Individuals who perceived higher levels of social support were less likely to report moderate or severe depressive and anxiety symptoms compared with those with no or minimal symptoms in fully adjusted models (Table 3). For example, every SD increase in social support lowered the odds for severe depression (OR, 0.53; 95% CI, 0.42-0.66) and anxiety symptoms (OR, 0.78; 95% CI, 0.62-0.98), by 47% and 22%, respectively. After changing the reference group to those with moderate symptoms, a dose-dependent response was also observed, with a stronger magnitude of association between social support and severe depressive symptoms than moderate depressive symptoms (OR, 0.79; 95% CI, 0.64-0.97; *P* < .025).

**Table 3.** Prospective Associations Between Social Support at 19 Years and Clinical Thresholds of Mental Health Problems at 20 Years (N = 1174), Imputed Values^a^

Model	OR (95% CI)			
	Depressive symptoms		Anxiety symptoms	
	Moderate	Severe	Moderate	Severe
Social support adjusted for sex	0.61 (0.53-0.70)	0.47 (0.39-0.58)	0.74 (0.62-0.88)	0.68 (0.56-0.83)
Social support adjusted for all other confounders^b^	0.67 (0.58-0.78)	0.53 (0.42-0.66)	0.84 (0.69-1.02)	0.78 (0.62-0.98)

Abbreviation: OR, odds ratio.

^a^ Data were compiled from the final master file of the Québec Longitudinal Study of Child Development (1998–2018), Québec Government, Québec Statistic Institute.

^b^ The fully adjusted model is additionally adjusted for mental health problems during adolescence (ages 15-17 years), including anxiety, depression, suicidal ideation and suicide attempts, attention deficit/hyperactivity-impulsivity, oppositional defiant and conduct symptoms as well as family characteristics in adolescence, including socioeconomic status, family functioning, and family structure (at age 17 years). Reference category: no or minimal symptoms for depressive and anxiety symptoms.

## Discussion

Using data from the QLSCD, we found that emerging adults who perceived a greater level of social support were less likely to report MHPs including suicidal ideation and attempts 1 year later, independent of previous MHPs and family characteristics. Benefits of social support were observed across the full spectrum of depressive and anxiety symptoms, including clinically increasing severe expression of these symptoms, and for both serious suicidal ideation and suicide attempts. Although social support could have protective benefits for both moderate and severe symptoms, the magnitude of this association was stronger for severe symptoms, indicating that social support may be particularly valuable for helping the most vulnerable individuals.

Consistent with previous studies,^6,36,37^ female emerging adults (vs male emerging adults) were found to perceive significantly more social support. Female individuals have been shown to invest in more emotionally involved interpersonal relationships compared with male individuals.^38,39^ The instrument used in this study^24^ (and in most studies evaluating perceived social support) focuses more heavily on an emotional component. This instrument may emphasize the type of support valued by female emerging adults, whereas their male counterparts may perceive and value support that emphasizes interactions such as engaging in shared activities.^36,40^ Thus, perceptual differences in social support may exist between sexes. Despite these differences, we found that individuals benefit from social support regardless of sex.

Previous evidence suggests a reciprocal cycle between levels of perceived social support and MHPs,^41^ thus, it is important to account for previous MHPs to better clarify whether social support per se is beneficial regardless of previous MHPs. We found significant negative correlations between adolescent MHPs and social support at age 19 years. Thus, individuals with worse mental health during adolescence also perceived lower levels of social support later on. We adjusted for a range of adolescent MHPs in all of the analyses to clarify the directionality of these associations. Unlike many previous studies^2^ that do not account for previous MHPs, we found that social support was associated with fewer mental health symptoms regardless of previous MHPs. The findings of the present study are similar to those of other longitudinal studies in emerging adulthood that have reported that young adults who enjoyed higher levels of social support reported fewer depressive symptoms after adjusting for baseline depressive symptoms. For instance, increases in social support were associated with shifts in depression trajectories.^15^ Yet another study that adjusted for baseline depressive symptoms found that social support was beneficial only in youth facing work and financial stress.^14^ However, a longitudinal study^18^ failed to detect an association of social support with trajectories of depressive symptoms, but this inconsistency may have occurred because of the study’s focus on parental support, which may be protective only when established earlier in life.^17^

We found that perceived social support was associated with fewer generalized anxiety symptoms, including severe anxiety. This finding is consistent with a prospective study that found that lower perceived parental social support in childhood was associated with a 4-fold higher risk of generalized anxiety disorder in emerging adulthood.^17^ However, previous MHPs were not accounted for and social support was based on retrospective reports. We believe that the findings of the present study support evidence from previous studies by showing that similar associations are observed between perceived social support and anxiety symptoms in emerging adulthood, while accounting for adolescent MHPs.

In addition, we found that social support was associated with less suicidal ideation in emerging adults. A previous study with female participants found that those who enjoyed higher levels of social support were more likely to be in remission from suicidal thoughts.^16^ Social support was also associated with fewer suicide attempts in emerging adults. The protective benefits of parental social support against suicide attempts during adolescence has been demonstrated.^42^ However, to our knowledge, this is the first study to highlight the association of perceived social support with fewer suicide attempts in emerging adulthood. A randomized clinical trial found that support from a Youth-Nominated Support Team (ie, characterized by psychoeducation and weekly emotional and instrumental support check-ins from supportive adults for 3 months) was associated with a reduced risk for suicide mortality in vulnerable adolescents aged 13 to 17 years.^43^ The limited prospective evidence using suicide attempts as an outcome is consistent with that of a cross-sectional study^44^ and points to the role of social support in suicide-related outcomes. The use of a social support measure with an unspecified source in the present study allowed respondents to reflect on a source of support available to them, rather than being restricted to reflect on a specific source. Results of this population-based study are also in line with those of previous studies reporting the protective benefits of social support for vulnerable groups (eg, adolescents who experience bullying,^45^ clinical populations,^46^ non-heterosexual populations^47^ and those exposed to higher levels of stress^14^).

### Strengths and Limitations

This study has several strengths, including the prospective design and the ability to adjust for a range of confounders (previous MHPs and family characteristics), which addresses to the limitations of reverse causality found in previous studies.^2^ Without adjusting for previous MHPs, we cannot effectively rule out the possibility that associations of social support with MHPs are better accounted for by the associations of MHPs with social support. Moreover, in contrast to several previous studies,^2,6^ social support was measured using a validated tool with excellent psychometric properties.^24^ Another major strength of the present study is that we investigated associations with clinically significant outcomes (ie, generalized anxiety and suicide-related outcomes)^48^ that are otherwise scarce in the social support literature, especially in the literature focusing on emerging adulthood.

This study also has some limitations. First, inherent to all longitudinal studies, attrition occurred among the most vulnerable individuals, such as those from low socioeconomic status and those displaying more externalizing symptoms at 29 months, which may have resulted in an underestimation of associations for these individuals. However, as the magnitude and patterns of associations were consistent across weighted, imputed, and maximum available samples, we believe that biases are likely to be minimal. Second, social support was self-reported, meaning that we captured perceptions (vs objectively received measures) of social support, which may have been influenced by mental health status at the time,^41^ which may result in an overestimation of effect sizes. However, in this population-based study, youth were the only informants given that they offer the most insight into their own suicidal behaviors^49^ and internalizing symptoms in comparison to parents or other informants. Although this study does not measure objective reports of social support or MHPs, future studies may consider using other informant sources to ascertain whether social support in itself is protective for mental health (ie, the objective measure of support), or whether the benefits of social support lie in the perception of it. Third, our study did not control for shared genetic vulnerability between both social support and MHPs.^50^ Although social support may be considered to be an environmental factor, research has pointed toward the role of genetic factors in social support.^51,52^ Using the twin study design, 2 studies found that social support and depression in adolescence were phenotypically associated, yet such associations were largely explained by genetic factors affecting both the perception of social support and depression. In contrast, a study using a discordant twin design in the Environmental Risk Longitudinal Twin Study (E-RISK) cohort found that the association of social support with psychotic symptoms was largely environmentally mediated.^53^ Thus, future studies should further investigate the magnitude of associations while considering a genetically informative design to untangle the unique advantages of social support. Finally, causality cannot be established by observational studies alone because the possibility that associations are better explained by unmeasured confounders cannot be ruled out. However, the findings of the present study are consistent with those of a randomized clinical trial reporting the protective benefits of social support in suicide-related outcomes.^43^

## Conclusions

Emerging adulthood is a transitional life period marked by a high prevalence of MHPs. This study provides evidence on the benefits associated with social support for MHPs and suicide-related outcomes during this life-period, even in individuals who experienced MHPs in an earlier stage of development. Therefore, this study raises awareness of the potential protective role of perceived social support for mental health during emerging adulthood and provides evidence for the importance of leveraging social support in treatment options while taking into consideration perceptions of support.

## Notes

Supplement. **eTable 1.** Comparisons of participants and non-participants on key variables based on unweighted values **eTable 2.** Correlations between social support at 19 years, mental health problems and suicide-related outcomes at 20 years and adolescent confounders from 15-17 years **eTable 3.** Prospective associations between social support at 19 years and mental health problems and suicide-related outcomes at 20 years; n=907, maximum available sample **eTable 4.** Prospective association between social support at 19 years and clinical threshold of MHPs at 20 years; n=907, maximum available sample
