# Supplementary material for: Association of Social Support During Adolescence With Depression, Anxiety, and Suicidal Ideation in Young Adults
Source: JAMA Netw Open. 2020 Dec 4;3(12):e2027491. doi: 10.1001/jamanetworkopen.2020.27491 (PMC7718598; doi:10.1001/jamanetworkopen.2020.27491)
Supplement: Supplement. — eTable 1. Comparisons of participants and non-participants on key variables based on unweighted values eTable 2. Correlations between social support at 19 years, mental health problems and suicide-related outcomes at 20 years and adolescent confounders from 15-17 years eTable 3. Prospective associations between social support at 19 years and mental health problems and suicide-related outcomes at 20 years; n=907, maximum available sample eTable 4. Prospective association between social support at 19 years and clinical threshold of MHPs at 20 years; n=907, maximum available sample [file jamanetwopen-e2027491-s001.pdf]

Supplemental Online Content

Scardera S, Perret LC, Ouellet-Morin I, et al. Association of social support during adolescence with depression, anxiety, and suicidal ideation in young adults. *JAMA Netw Open*. 2020;3(12):e2027491. doi:10.1001/jamanetworkopen.2020.27491

eTable 1. Comparisons of participants and non-participants on key variables based on unweighted values
eTable 2. Correlations between social support at 19 years, mental health problems and suicide-related outcomes at 20 years and adolescent confounders from 15-17 years
eTable 3. Prospective associations between social support at 19 years and mental health problems and suicide-related outcomes at 20 years; n=907, maximum available sample
eTable 4. Prospective association between social support at 19 years and clinical threshold of MHPs at 20 years; n=907, maximum available sample

This supplemental material has been provided by the authors to give readers additional information about their work.

© 2020 Scardera S et al. *JAMA Network Open.*

eTable 1: **Comparisons of participants and non-participants on key variables based on unweighted values**[a]

	participants(n=1174)	non-participants(n=946)	p-value
**Child characteristics**			
Male, No. (%)	494(42.1)	586(61.9)	<.001
Birth weight (kg), mean (SD)	3.40(50)	3.40(.50)	.971
Non-Canadian, No., %	303(25.9)	297(31.7)	.004
Internalizing behaviors at 29 months[b]	0.90(1.21)	0.89(1.23)	.732
Externalizing behaviors at 29 months[c]	4.91(2.49)	5.24(3.00)	.014
**Family Characteristics**			
Maternal age at birth in years, mean (SD)	29.18(5.47)	28.51(5.46)	.004
Maternal depression[d], mean (SD)	1.32(1.29)	1.50(1.40)	.002
Non-intact family (single or blended), No. (%)	190(16.2)	216(23.0)	<.001
Family socioeconomic status[e]	0.17(.96)	-0.19(1.00)	<.001

Data were compiled from the final master file of the Québec Longitudinal Study of Child Development (1998-2018), Québec Government, Québec Statistics Institute.
[a]Variables were measured when the child was 5 months of age, unless otherwise indicated.
[b]Assessed at 29 months, missing values were replaced with 17 months; 6 items from the Behavior Questionnaire(32, 49) (e.g., is too fearful or anxious), scores range from 0-8.
[c]Assessed at 29 months, missing values were replaced with 17 months; 10 items from the Behavior Questionnaire(32, 49) (e.g., cannot sit still, is agitated), scores range from 0-18.
[d]Assessed using a shortened version (12 items) of the Center for Epidemiologic Studies-Depression(25). Scores were standardized to range from 0-10.
[e]z-scores reflecting an aggregate of parental educational level, annual gross income and parental education level(31).

© 2020 Scardera S et al. *JAMA Network Open.*

eTable 2: **Correlations between social support at 19 years, mental health problems and suicide-related outcomes at 20 years and adolescent confounders from 15-17 years†**

	Social Support	Depression	Anxiety	Ideation	Attempts
Family Characteristics 15-17y					
Sex	**.09**	**.16**	**.22**	-.01	-.01
Socio-economic status	**.12**	-.04	**-.06**	-.01	-.03
Hostile-Reactive Parenting	-.04	.01	.04	.01	-.03
Family Structure at 17y	**-.08**	.05	.05	.05	**.07**
Family Functioning	**-.08**	**.07**	**.09**	.03	-.01
Mental Health 15-17y					
Anxiety	**-.16**	**.38**	**.42**	**.15**	**.13**
Depression	**-.20**	**.45**	**.40**	**.18**	**.17**
Conduct	**-.14**	**.14**	**.11**	**.11**	**.10**
Oppositional/Defiant	**-.14**	**.23**	**.22**	**.13**	**.11**
Inattention/Hyperactivity	**-.12**	**.27**	**.26**	**.13**	**.11**
Suicide Ideation	**-.10**	**.13**	**.17**	**.24**	**.19**
Suicide Attempts	-.05	**.08**	**.11**	**.15**	**.11**

Data were compiled from the final master file of the Québec Longitudinal Study of Child Development (1998-2018), Québec Government, Québec Statistics Institute.
†based on maximum n available ranging from 907 to 1174.
All continuous confounder scores measured in adolescence were standardized into z-scores and averaged across ages 15 and 17 years. Bolded estimates are statistically significant; p<.05.

© 2020 Scardera S et al. *JAMA Network Open.*

eTable 3: Prospective associations between social support at 19 years and mental health problems and suicide-related outcomes at 20 years; *n*=907, maximum available sample.

	Depressive symptoms		Anxiety symptoms		Suicide ideation	Suicide attempts
	β	*p*-value	β	*p*-value	OR (95% CI)	OR (95% CI)
Social support						
Adjusted for sex	-0.30	<.001	-0.17	<.001	0.57(0.48-0.68)	0.57(0.45-0.73)
Fully adjusted	-0.22	<.001	-0.09	.004	0.62(0.52-0.73)	0.61(0.46-0.81)

Data were compiled from the final master file of the Québec Longitudinal Study of Child Development (1998–2018), Québec Government, Québec Statistic Institute.
OR=odds ratio; 95%CI= 95% confidence intervals.
The fully adjusted model is additionally adjusted for mental health problems in adolescence (15-17 years) including anxiety, depressive, suicidal ideation/attempts, attention deficit hyperactivity-impulsivity, oppositional defiant and conduct symptoms; family characteristics in adolescence (15-17 years) including socioeconomic status and family functioning and family structure (17 years)
P-values for socialsupport*sex interactions were for depressive (*p*=.942) and anxiety symptoms (*p*=.502), suicidal ideation (*p*=.826) and attempts (*p*=.228).

© 2020 Scardera S et al. *JAMA Network Open.*

eTable 4: Prospective association between social support at 19 years and clinical threshold of MHPs at 20 years; *n*=907, maximum available sample.

	OR (95% CI)			
	Depressive symptoms		Anxiety symptoms	
	Moderate	Severe	Moderate	Severe
Social support				
Adjusted for sex	0.62(0.54-0.71)	0.46(0.38-0.57)	0.71(0.60-0.85)	0.65(0.53-0.80)
Fully adjusted	0.72(0.61-0.85)	0.56(0.44-0.70)	0.86(0.69-1.06)	0.75(0.59-0.96)

Data were compiled from the final master file of the Québec Longitudinal Study of Child Development (1998–2018), Québec Government, Québec Statistic Institute.
OR=odds ratio; 95%CI= 95% confidence intervals.
The fully adjusted model is additionally adjusted for mental health problems in adolescence (15-17 years) including anxiety, depressive, suicidal ideation/attempts, attention deficit hyperactivity-impulsivity, oppositional defiant and conduct symptoms; family characteristics in adolescence (15-17 years) including socioeconomic status, family functioning and family structure (17 years)
Reference categories: no/minimal symptoms for depressive/anxiety symptoms.
P-values for socialsupport*sex interactions were for moderate depressive (p=.105) and anxiety symptoms (p=.471) and for severe depressive (p=.955) and anxiety symptoms (p=.281).

© 2020 Scardera S et al. *JAMA Network Open.*
